# Unexpected larger distribution of paleogene stem-rollers (AVES, CORACII): new evidence from the Eocene of Patagonia, Argentina

**DOI:** 10.1038/s41598-020-80479-8

**Published:** 2021-01-14

**Authors:** Federico J. Degrange, Diego Pol, Pablo Puerta, Peter Wilf

**Affiliations:** 1 Centro de Investigaciones en Ciencias de la Tierra (CICTERRA), UNC, CONICET, Avenida Vélez Sársfield 1611, X5016GCA Córdoba, Argentina; 2 Museo Paleontológico Egidio Feruglio-CONICET, Av. Fontana 140, U9100GYO Trelew, Chubut Argentina; 3 Department of Geosciences and Earth and Environmental Systems Institute, Pennsylvania State University, University Park, PA 16802 USA

**Keywords:** Palaeontology, Phylogenetics

## Abstract

Here we present the first record of a stem-Coracii outside the Holarctic region, found in the early Eocene of Patagonia at the Laguna del Hunco locality. *Ueekenkcoracias tambussiae* gen. et sp. nov. consists of an incomplete right hind limb that presents the following combination of characters, characteristic of Coracii: relatively short and stout tibiotarsus, poorly developed crista cnemialis cranialis, short and wide tarsometatarsus, with the tuberositas m. tibialis cranialis located medially on the shaft, and curved and stout ungual phalanges. Although the presence of a rounded and conspicuous foramen vasculare distale and the trochlea metatarsi II strongly deflected medially resemble Primobucconidae, a fossil group only found in the Eocene of Europe and North America, our phylogenetic analysis indicates the new taxon is the basalmost known Coracii. The unexpected presence of a stem-Coracii in the Eocene of South America indicates that this clade had a more widespread distribution than previously hypothesized, already extending into the Southern Hemisphere by the early Eocene. *Ueekenkcoracias tambussiae* represents new evidence of the increasing diversity of stem lineages of birds in the Eocene. The new material provides novel morphological data for understanding the evolutionary origin and radiation of rollers and important data for estimates of the divergence time of the group.

## Introduction

Coracii (rollers) is a group of Neognathae that includes colorful and excellent-flying birds, with large heads and stout beaks that live in diverse habitats ranging from open savannas to river valleys^1^. Although highly diverse with a mainly paleotropical distribution today, the fossil record of Coracii shows that early members of this group were present in Europe and North America during the Eocene; however, their presence in South America has not been reported. The Eocene Coraciiformes have been interpreted as basal rollers (that have recently been placed in a separate clade, the Coracii Wetmore and Miller^2^, sensu.^3,4^) and include *Primobucco* and *Paracoracias* from the early Eocene portion of the Green River Formation in western North America^5–8^, *Septencoracias* from the early Eocene Fur Formation of Denmark^9^, *Eocoracias* from the early Eocene Messel locality^10^, and *Geranopterus* from the late Eocene and Miocene of Europe^10^.

The fossil record of Eocene continental birds from South America is notably scarce, and so far most of the remains correspond to extinct lineages (e.g., Presbyornithidae and Phorusrhacidae) or are too fragmentary to make precise systematic assignments^11^. The early Eocene Huitrera Formation^12^ at the Laguna del Hunco locality of Chubut Province, Argentina includes the remains of a fossil caldera lake that is renowned for its diverse and exceptionally well-preserved fossil plant diversity^13–15^. Described vertebrates from this locality include only finely preserved fishes and frogs^16–18^. Here we report a new bird species recently found at the Laguna del Hunco locality that is represented by a right hind limb and interpreted as a stem-Coracii. This new taxon expands into South America the geographic distribution of fossil birds related to old-world extant rollers.

## Systematic paleontology

Class Neornithes Linnaeus, 1758.

Subclass Neognathae Pycraft, 1900.

Clade Coracii Wetmore and Miller, 1926.

*Ueekenkcoracias tambussiae* gen. et sp. nov.

### Etymology

The generic name is from the native Tehuelche word *ueekenk*, meaning “outsider” in relation to its unexpected presence in South America, and the genus name ‘*Coracias*’. The species name honors Claudia Patricia Tambussi, whose contributions to paleornithology in the last three decades have fostered our understanding of the diversity and evolution of fossil birds in South America.

### Holotype

**Figure 1:**
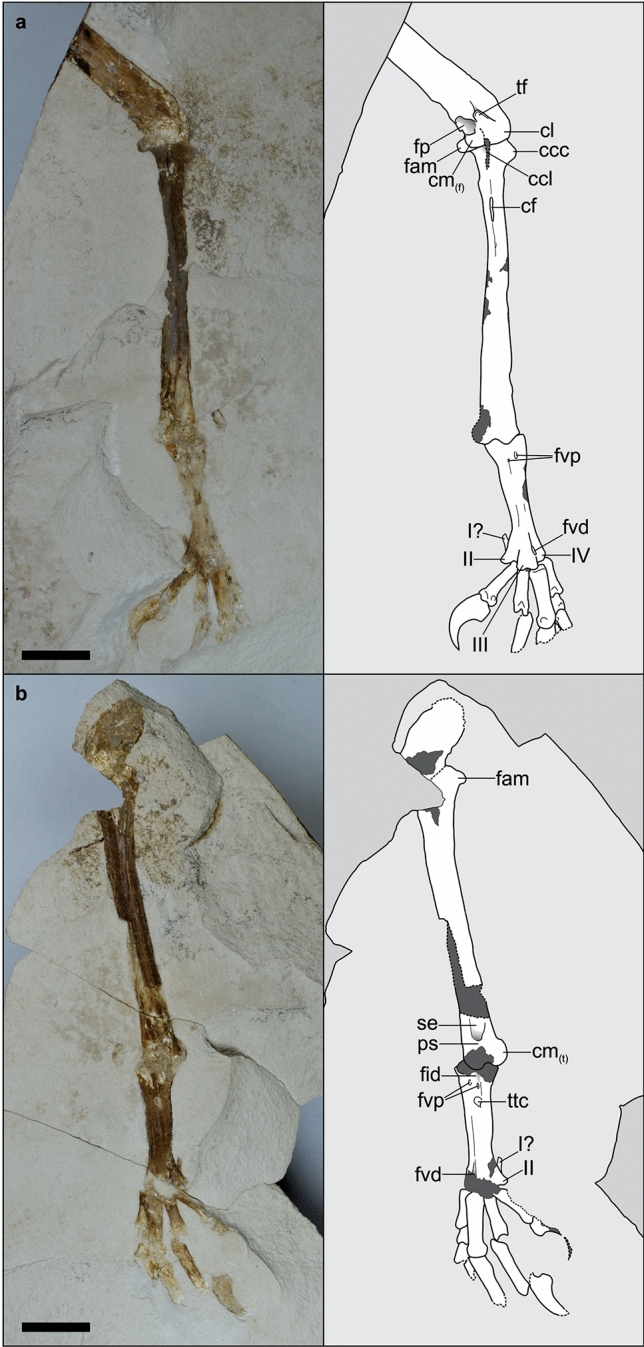
*Ueekenkcoracias tambussiae* gen. et sp. nov., holotype MPEF-PV 10991. Photograph (left) and interpretative drawing (right) of slabs (**a**,**b**). *ccc* crista cnemialis cranialis, *ccl* crista cnemialis lateralis, *cf* crista fibularis, *cl* condylus lateralis, *cm*_*(f)*_ condylus medialis (femur), *cm*_*(t)*_ condylus medialis (tibiotarsus), *fam* facies articularis medialis, *fid* fossa infracotylaris dorsalis, *fp* fossa poplitea, *fvd* foramen vasculare distale, *fvp* foramina vascularia proximalia, *I?* metatarsi I?, *II–IV* trochlea metatarsi II–IV, *ps* pons supratendineus, *se* sulcus extensorius, *tf* trochlea fibularis, *ttc* tuberositas m. tibialis cranialis. Scale = 1 cm.

**Figure 2:**
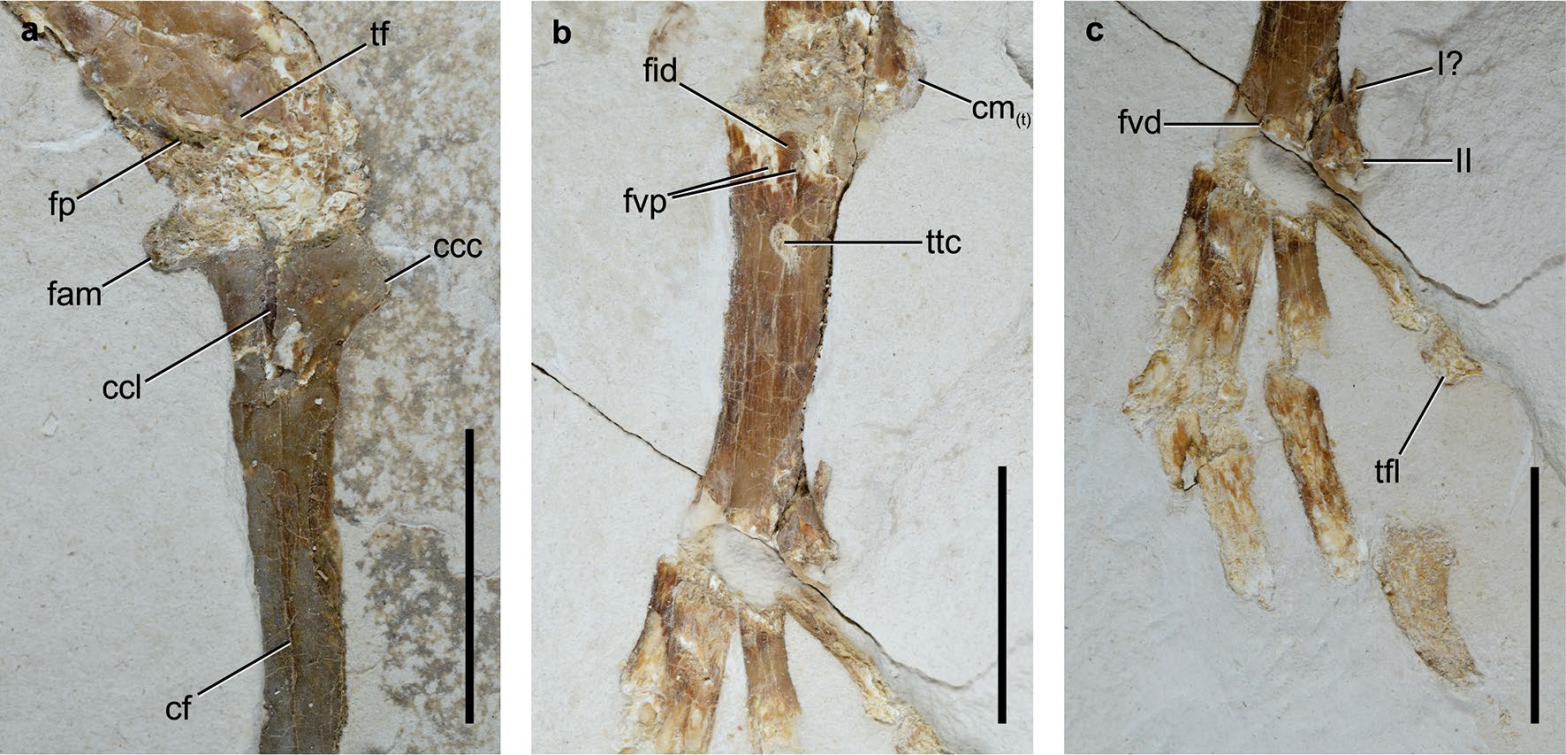
*Ueekenkcoracias tambussiae* gen. et sp. nov., holotype MPEF-PV 10991. (**a**) detail of the proximal end of tibiotarsus; (**b**) tarsometatarsus; (**c**) detail of the foot. *ccc* crista cnemialis cranialis, *ccl* crista cnemialis lateralis, *cf* crista fibularis, *cm*_*(t)*_ condylus medialis (tibiotarsus), *fam* facies articularis medialis, *fid* fossa infracotylaris dorsalis, *fp* fossa poplitea, *fvd* foramen vasculare distale, *fvp* foramina vascularia proximalia, *I?* metatarsi I?, *II* trochlea metatarsi II, *tf* trochlea fibularis, *tfl* tuberculum flexorium, *ttc* tuberositas m. tibialis cranialis. Scale = 1 cm.

MPEF-PV 10991, incomplete right hind limb, preserved in two slabs as part and counterpart (Figs. 1, 2).

### Locality and horizon

Laguna del Hunco locality, quarry LH27, Huitrera Formation, early Eocene (Ypresian), ca. 52.2 Ma based on ^40^Ar–^39^Ar dates and paleomagnetic stratigraphy ^13–15,19^, Chubut Province, Argentina.

### Diagnosis

A large representative of stem-Coracii, with a robust femur and a stout tibiotarsus, the facies articularis medialis is strongly projected caudally, the crista cnemialis cranialis is triangular shaped, the pons supratendineus is directed latero-medially, located in the median axis of the shaft, and proximally to the condyli, on the shaft, the trochleae metatarsorum II and IV have the same distal extension, the trochlea metatarsi II is medially deflected.

### Description

The material consists of an incomplete and crushed right hind limb (Fig. 1). The distal half of the femur has been preserved, and the tibiotarsus, tarsometatarsus, and pes are mostly complete, although some elements are incompletely exposed or partially damaged.

The preserved portion of the femur shows the corpus femoris is robust and straight, as is typical of Coracii, and the condyli are poorly projected distally. The trochlea fibularis is flat, and the fossa poplitea is small.

As in all Coracii, the tibiotarsus is relatively short and stout (proportionally shorter in *Eocoracias*). The facies articularis medialis is strongly projected caudally (vs. poorly projected in Leptosomidae, Coraciidae, Meropidae, Momotidae and Alcedinidae). The crista cnemialis lateralis is not preserved, and the crista cnemialis cranialis is triangular shaped and small (Fig. 2a), but more developed than in *Eocoracias* and *Paracoracias*^8^. The crista cnemialis cranialis of *Ueekenkcoracias*, however, is not as reduced as in Coraciidae, Alcedinidae and Meropidae. The diaphysis of the tibiotarsus is relatively stout (vs. slender in Momotidae and Todidae^20,21^), and slightly curved laterally (rather than straight as in Alcedinidae). The crista fibularis is distally located (vs. proximally located in Momotidae). The pons supratendineus has a transverse disposition, as in Geranopteridae and similar to Momotidae, and it is located medially on the shaft as in Coraciidae and Brachypteraciidae, and proximally to the condyli, on the shaft (more proximally than in Momotidae). In Leptosomidae, the pons supratendineus is markedly stout, and strongly slanted distolaterally, in Coraciidae the pons is small, and slightly slanted distolaterally, placed between the proximal edges of the condyles, as in Geranopteridae. The distal epiphysis widens distally (i.e., both condyli are more separated from each other), as in Coraciidae, Brachypteraciidae, Todidae^20^, but not as much as in Leptosomidae^22^, a family distantly related to rollers^3,23^. The condylus medialis is strongly projected caudally, similar to the condition of Alcedinidae, and contrary to Momotidae.

The tarsometatarsus of *Ueekenkcoracias* is short and wide (Fig. 2b), resembling the condition of Primobucconidae^24^, *Parvicuculus*^25,26^, Eocoracidae^23^, Alcedinidae, and Meropidae, measuring less than half the length of the tibiotarsus (Table 1), but different from the more elongated tarsometatarsus of Geranopteridae, and evidently contrary to the condition observed in Brachypteraciidae, Todidae, and Momotidae, all of which have greatly elongated and slender tarsometatarsus. The proximal end is wider than the diaphysis, particularly on its medial side (vs. strongly expanded lateromedially in Leptosomidae and Todidae). Although poorly preserved, the eminentia intercotylaris is low (similar to Coraciidae, and contrary to Alcedinidae and Brachypteraciidae). The fossa infracotylaris dorsalis is rounded and well-marked, although shallow, which is contrary to what is observed in Leptosomidae, Coraciidae and *Septencoracias*^9^. The foramina vascularia proximalia are conspicuous, with the lateral foramen more proximally located than the medial (whereas they are located at the same level in Todidae^20^). As in Primobucconidae and other members of Coracii, and Meropidae, the tuberositas m. tibialis cranialis is located on the medial margin of the shaft, distal to the foramina vascularia proximalia^8,9,24^, forming an elongated scar with a medial tubercle. The facies dorsalis is smooth and flat distally as in Geranopteridae, Coraciidae, and the Alcedinidae *Halcyon*, but contrary to Leptosomidae^27^ and other Alcedinidae such as *Dacelo* and *Ceryle* that present a well-marked sulcus extensorius. The trochleae metatarsorum II and IV seems to have the same distal extension, being less than that of the trochlea metatarsi III. In Leptosomidae the trochlea metatarsi II reaches farther distally than trochlea metatarsi IV^22^, and among Coracii, the disposition of the trochleae metatarsorum is highly variable: the trochlea metatarsi IV reaches farther distally than trochlea metatarsi II in Coraciidae^28^, the trochlea metatarsi IV reaches almost as far distally as the trochlea metatarsi III in *Eocoracias*^23^, the trochleae metatarsorum II and IV are only slightly shorter than the trochlea metatarsi III in Todidae^21^; and the trochleae metatarsorum III and IV are equally extended distally in Geranopteridae^23^ and Momotidae. The trochlea metatarsi II is medially deflected, as in *Primobucco* and Alcedinidae, but less than in Geranopteridae. The trochlea metatarsi IV is narrow and distally directed. The foramen vasculare distale is rounded, large (small in *Eocoracias* and Momotidae, absent in Meropidae) and very conspicuous, as in Primobucconidae^24^ (not discernible in *Paracoracias* according to Ref.^8^).

**Table 1:** Measuremetns (mm) in *Ueekenkcoracias tambussiae* gen. et sp. nov. (MPEF-PV 10991) compared with other Coracii.

	TL	TTL	TPW	TDW
*Ueekenkcoracias tambussiae*	45	19.3	6.3	6.3
*Primobucco mcgrewi*^a^	26.7	13.1	–	–
*Primobucco perneri*^a^	20.9–24	11.5–13.1	–	–
*Septencoracias morsensis* MGUH.VP 9509	30.9^a^	15.5^a^	7.6	6.7
*Eocoracias brachyptera* SMNK.PAL.2663	41.3^a^	18.2^a^	–	–
*Paracoracias occidentalis* AMNH FARB 30572	39.7^a^	19.2^a^	–	–
*Coracias garrulus* ZMB 4467	43.6	22.8	4.9	4.8

*TL* tibiotarsus length, *TTL* tarsometatarsus length, *TDW* tarsometatarsus distal width, *TPW* tarsometatarsus proximal width.

^a^From Ref.^9^.

The foot of *Ueekenkcoracias* has an anisodactyl toe arrangement (Fig. 2c), contrary to Leptosomidae, which has a zygodactyl-like arrangement^27^. In contrast to Primobucconidae, the pedal phalanges are short and robust, contrary to the phalanges of Leptosomidae, Todidae, Momotidae and Coraciidae that are elongated. Also, the unguals of Ueekenkcoracias present a very well-marked furrow on the sides, as in Alcedinidae. As in *Septencoracias* and Todidae, and contrary to *Paracoracias* and *Primobucco*, the first phalanx of the hallux is elongated. Nevertheless, in Todidae the first phalanx is the longest of all^21^, which is not the case for *Ueekenkcoracias*. The ungual phalanx I is stout, apparently lacking sulci, but with a well-developed tuberculum flexorium that is ventrally directed (similar to Alcedinidae and Leptosomidae). The tuberculum extensorium is small, although conspicuous (well-developed in Todidae). Regarding the second toe, the second phalanx is markedly more elongated than the first, as in *Septencoracias*. The first phalanx of the third toe is robust and longer than the first phalanx of the first toe. The ungual phalanx II is stout and poorly curved. The fourth toe has the first phalanx longer than the rest, which are subquadrangular in shape. The configuration of phalanges resembles more the condition observed in *Paracoracias* with relatively short phalanges than the condition of *Septencoracias* and Alcedinidae, which have more elongated and slender phalanges.

### Phylogenetic relationships

**Figure 3:**
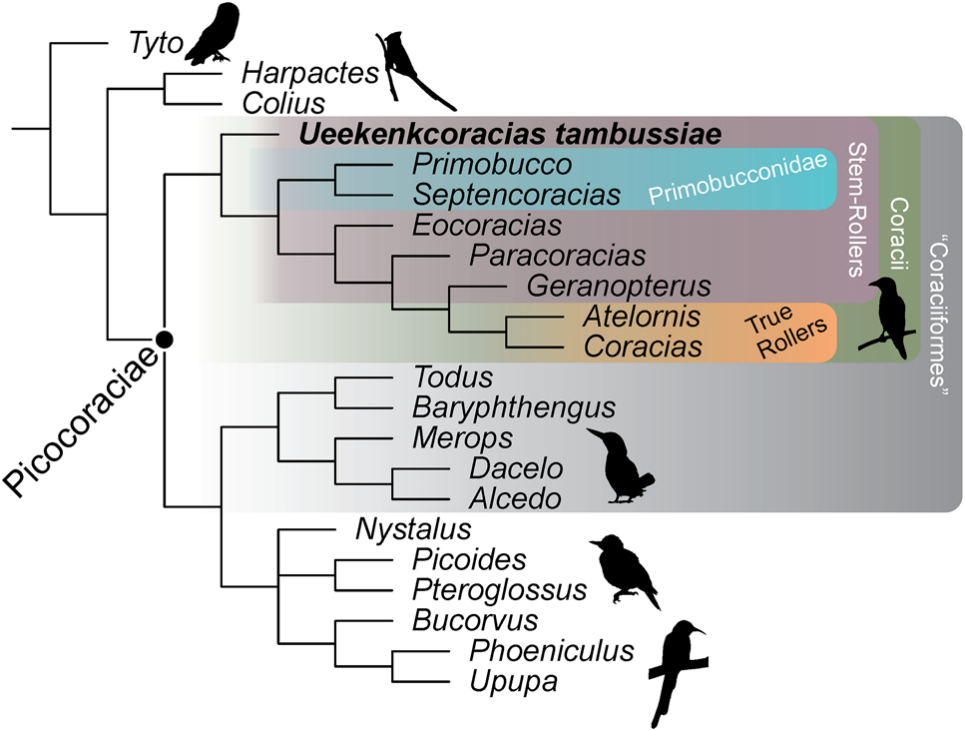
Phylogenetic relationships of *Ueekenkcoracias tambussiae* gen. et sp. nov. Bird silhouettes were taken from PhyloPic (PhyloPic—Free Silhouette Images of Life Forms).

The parsimony phylogenetic analysis resulted in two most parsimonious trees of 205 steps (see “Methods” and Supplementary Information), generating a well resolved strict consensus tree (Fig. 3). The results of alternative methods of analysis resulted are identical in terms of the phylogenetic position of *Ueekenkcoracias* (see Supplementary Information). The position of *Ueekenkcoracias* with other Coracii is supported by the presence of an enlarged foramen vasculare distale, a sharp, oblique ridge on the distal margin of the tarsometatarsus, and a marked groove proximal to the foramen vasculare distale. Nodal support for this position is low (Bremer = 2; jackknife = 60; see Supplementary Information, because the incompleteness of the new specimen precludes placing it with high confidence. However, alternative positions with other clades of Picocoraciae (e.g., Alcedinidae, Meropidae, Todidae) require at least four extra steps (see Supplementary Information). *Ueekenkcoracias* is placed as the earliest branch of the clade Coracii, being the sister taxon of Primobucconidae and other Coracii. This position is supported by the absence of two derived features that are shared by all other Coracii except for *Ueekenkcoracias*: trochlea metatarsi IV rotund in lateral view and reaching as far distally as trochlea metatarsi III and tarsometatarsus with deep dorsal infracotylar fossa.

## Discussion

The presence of a stem-Coracii in South America is surprising, but the phylogenetic results and the presence of unique derived features of this clade, such as a rounded and conspicuous foramen vasculare distale and the trochlea metatarsi II strongly deflected medially, suggests *Ueekenkcoracias* is related to stem-Coracii from the Eocene of Europe and North America (e.g., Primobucconidae, *Eocoracias*, *Paracoracias*). The new specimen is the oldest record of Coracii outside Europe and North America, indicating that this clade had a more widespread distribution than previously hypothesized and extending its geographical range into the Southern Hemisphere by the early Eocene.

The unexpected presence of Old World birds outside their extant distribution has been reported in other instances during the Paleogene. However, these records mainly consist of stem groups recorded in North America. Such is the case, among others, of *Tsidiiyazhi abini*, a stem-Coliiformes from the early Paleocene of New Mexico^29^, *Sandcoleus copiosus*, a stem-Coliiformes from the early Eocene of Wyoming^6^, *Foro panarium*, a stem-Musophagiformes, and *Celericolius acriala* a stem-Coliiformes from the early Eocene of Wyoming^30,31^.

Close affinities of *Ueekenkcoracias* to these other stem-groups are unlikely based on its tarsometatarsus morphology. The tarsometatarsus of Coliiformes is markedly more elongated and slenderer, with the trochleae metatarsorum II and IV reaching almost the distal extension of trochlea metatarsi III^32^, and the trochlea metatarsi IV is more laterally disposed than in the fossil presented here. Moreover, the phalanges in Coliiformes are slenderer and, particularly, the first phalanx of the second toe is much shorter than in *Ueekenkcoracias* (e.g., Refs.^6,32^); ungual phalanges are also slenderer in Coliiformes. In comparison with stem-Musophagiformes, the main differences include a shorter tibiotarsus and tarsometatarsus, a less developed crista cnemialis cranialis, and trochleae metatarsorum that are less spread than in *Foro*^30,33^.

A bird from the early Eocene of Europe and North America that has been related to Primobucconidae is *Parvicuculus minor*. However, the phylogenetic placement of that bird remains controversial^25,26^. *Ueekenkcoracias* shares with *Parvicuculus* a short and robust tarsometatarsus and the presence of a marked foramen vasculare distale, all features shared also with Primobucconidae. Moreover, *Ueekenkcoracias* has a shallower fossa infracotylaris dorsalis and a more medially located tuberositas m. tibialis cranialis. Also, the trochlea metatarsi IV in *Parvicuculus* is more proximally located than trochlea II (equally extended distally in *Ueekenkcoracias*) and more extended laterally (see Ref.^26^).

Coracii is the clade that includes extant rollers (Coraciidae and Brachypteridae) and their fossil relatives, such as Primobucconidae, Eocoraciidae, *Paracoracias* and Geranopteridae^8,9^. Traditionally, Coracii was included within Coraciiformes. However, there is still debate about the monophyly of Coraciiformes, the clade that according to some authors (e.g., Refs.^10,24,34,35^) includes other extant families such as Alcedinidae, Momotidae, Todidae, and Meropidae (Alcediniformes according to Ref.^36^) together with Coracii. “Coraciiformes” have a relatively short tibiotarsus, with reduced cristae cnemiales, relatively short tarsometatarsus, widened in some groups (such as Alcedinidae), with the tuberositas m. tibialis cranialis medially located and a hypotarsus subtriangular or subrectangular with sulci and foramina. Among “Coraciiformes,” a particular combination of characters of *Ueekenkcoracias* may suggest affinities with kingfishers (Alcedinidae), such as the presence of a femur with straight diaphysis, the condylus medialis of the tibiotarsus caudally projected, anisodactyl foot, short and wide tarsometatarsus with a widened proximal epiphysis (especially medially), foramina vascularia proximalia located at the same level proximally, tuberositas m. tibialis cranialis located medially on the diaphysis, trochlea metatarsi II strongly deflected medially, and presence of a large and rounded foramen vasculare distale. However, contrary to Alcedinidae, *Ueekenkcoracias* has a more developed eminentia intercotylaris, the first phalanx of the hallux is not medially expanded (an apomorphic condition of Alcedinidae according to Ref.^36^), and the phalanges are shorter and stouter than those of the majority of the Alcedinidae (in which they are usually slender, although its length varies among genera: long in *Dacelo*, short in *Megaceryle*). If *Ueekenkcoracias* is a stem-kingfisher, this will imply a much earlier origin of the group, which has a scarce fossil record and an origination time estimated at ca. 37.5 Ma^35,37^, almost 15 Myr after the *Ueekenkcoracias* record.

According to McCullough et al*.*^35^, Coraciiformes (i.e., Coracii + Alcediniformes) have a Palaeartic origin. They achieved this conclusion including some fossils of this large group in their analysis (e.g., Refs.^9,28^). If Coraciiformes are monophyletic as proposed by those authors, then the clade seems to have conquered South America at least in four pulses: a lineage of stem-Coracii during the early Eocene and crown-Coraciiformes during the late Miocene-early Pleistocene (Alcedinidae twice in the late Miocene and Pleistocene, and Momotidae in the Pliocene^26,28^). Otherwise, if Coraciiformes is paraphyletic, as most morphological-based analysis indicate^8^, Coraciiformes sensu stricto^36^ reached South America before the Alcediniformes Alcedinidae, and Momotidae. From these groups, clearly, the lineage of stem-Coracii did not succeed, and the present distribution of Coraciiformes sensu lato in the world seems to be explained by the deterioration of warm climates at middle and high latitudes after the early Eocene climatic optimum, resulting in their extant pantropical distribution (e.g., Ref.^8^).

The rich fossil plant assemblage at the Laguna del Hunco locality represents the environmental conditions in Patagonia during the early Eocene climatic optimum. Recent paleoenvironmental and floristic comparisons indicate that the closest modern analogs for the Laguna del Hunco flora are the Malesian lower-montane tropical, everwet rainforests^15^, where diverse extant “Coraciiformes” exist today. The Laguna del Hunco paleoenvironment resembles that reported for Holarctic stem-Coracii, such as Primobucconidae^7–9^ in being a frost-free, warm lakeshore environment, although they differ in their seasonality (seasonally dry vs. everwet). In fact, the age of the Laguna del Hunco biota, ca. 52.2 Ma^13–15,19^, is very similar to that of the Fossil Butte Member of the Green River Formation (51.66 ± 0.09 Ma), the source of *Primobucco mcgrewi*^7,38^. Although *Ueekenkcoracias* is not the oldest stem-Coracii, due to the age of *Septencoracias* at ca. 54 Ma, the new taxon presented here provides important data for understanding the early biogeographic history of Coracii during the early Eocene.

According to Claramunt and Cracraft^34^, modern ornithofaunas are the result of recurrent dispersal events using two main routes: one connecting South America with the Old World via North America and another one connecting South America with Australia and New Zealand through Antarctica. Those authors^34^ postulated that “Coraciiformes” (i.e., Coracii + Alcediniformes) colonized the Paleotropics from North American ancestors. Given the presence of many of these lineages in the early Paleogene of Europe, they inferred that “Coraciiformes” reached the western Palearctic through a North Atlantic corridor before ~ 52 Ma. Although the ‘North American Gateway’ hypothesis explains well the origins of Musophagiformes^30^ and Coliiformes^29^, it does not explain the current fossil record of Coracii, due to the presence of *Ueekenkcoracias* as the basalmost stem-Coracii in the early Eocene of South America.

The arrival of a stem-Coracii lineage to South America may have occurred from North America if this clade has the biogeographic origin postulated by Ref.^34^, which would also open a second possible dispersal route from North America to Africa (in addition to the European route^34^). Alternatively, the stem-Coracii may have arrived in South America from Africa if the latter continent is the biogeographic origin for the group, as postulated by the biogeographic analysis of Ericson^39^. Interestingly, this scenario is also compatible with the Afrotropical ancestral area reconstruction for several basal nodes of “Coraciiformes” + Piciformes in the analysis of Ref.^34^. This possibility is based on ancestral areas from biogeographic analyses, but unfortunately no records of stem-Coracii have been found yet in Africa.

In either scenario, a dispersal route for stem-Coracii across the South Atlantic between Africa and South America fits the biogeographic pattern^40,41^ recognized for several vertebrate groups in the Eocene, including mammals (e.g., rodents^42^, primates^43^), birds (e.g., Phorusrhacidae^44^, Opisthocomiformes^45^), and reptiles^46,47^. Rafting events or even the possible presence of island chains^40^ have been advocated as explanations for these faunal interchanges between Africa and South America during the Paleogene^40–47^. It is possible that small stem-Coracii also dispersed across the Southern Atlantic, either by flying, rafting, or island hopping while Africa was still relatively close to NE South America.

## Conclusions

*Ueekenkcoracias tambussiae* provides evidence for the existence of a previously unrecognized group of birds in the early Eocene of South America and adds new information to the poorly known South American Paleogene ornithofaunas^11^. The new species is interpreted as a stem-Coracii, a clade that appears to have had a much wider distribution than previously thought. *Ueekenkcoracias* inhabited everwet rainforests during the Eocene in Patagonia. Moreover, *Ueekenkcoracias* provides new evidence that “Coraciiformes” (i.e., Coracii + Alcediniformes) have reached South America at least four times, once in the Paleogene and three times in the Neogene and early Pleistocene (see Refs.^35,37^).

*Ueekenkcoracias tambussiae* adds to the increasing diversity of stem bird lineages recorded in the Eocene. Although *Ueekenkcoracias* is not the oldest record of Coracii, it represents the earliest divergent lineage of Coracii because it is positioned as the sister group of primobucconids and more derived taxa. Low branch support in our phylogenetic analysis is due to the incompleteness of the fossil, and additional fossil material will help to clarify the relationships of the fossil to other “Coraciiformes”. The new discovery has a profound impact on the early biogeographical patterns of Coracii. The presence of the basalmost stem-roller *Ueekenkcoracias* in the Eocene of South America could be the result of dispersal events from North America or alternatively from Africa, resembling the biogeographic history proposed for other Paleogene vertebrates.

## Methods

### Institutional abbreviations

AMNH, American Museum of Natural History, New York, U.S.A.; CIT-O, Avian Osteological Collection of the Centro de Investigaciones en Ciencias de la Tierra (CICTERRA), Córdoba, Argentina; MGUH, Geological Museum, Copenhagen, Denmark; MLP, Museo de la Plata, La Plata, Argentina; MPEF-PV, Museo Egidio Feruglio, Trelew, Argentina; SMNK, Staatliches Museum für Naturkunde, Karlsruhe, Germany; USNM, Smithsonian National Museum of Natural History, Washington D.C., U.S.A.; ZMB, Museum für Naturkunde, Berlin, Germany.

### Material examined

*Ceryle torquata* CIT-O6; *Chloroceryle americana* MLP 5; *Chloroceryle amazona* MLP 90; *Coracias garrulus* ZMB 4467, ZMB 4469, ZMB 44211; *Dacelo leachii* ZMB 10; *Dacelo novaeguineae* ZMB 4437; *Eurystomus glaucurus* ZMB 4462; *Foro panarium* USNM 336261; *Halcyon chelicuti* ZMB 4436; *Halcyon smyrnensis* ZMB 32418; *Leptosomus discolor* ZMB 4473; *Momotus momota* CIT-O152; *Paracoracias occidentalis* AMNH FARB 30572. Observations of the taxa Meropidae, Todidae, *Parvicuculus*, Primobucconidae, Eocoracidae, Geranopteridae, and Brachypteraciidae were made from the original figures and descriptions in the published literature.

### Phylogenetic analysis

The primary purpose of our phylogenetic analysis was to test the affinities of the new taxon with Coracii (or Coraciiformes sensu stricto according to Refs.^24,36^). The phylogenetic position of the new taxon was evaluated using the character-taxon matrix of Ref.^9^, which includes 78 characters, an ingroup of 18 extant and extinct Picocoraciae, two Coliiformes, and *Tyto* (Strigiformes) as the outgroup. Following^9^, characters 13 and 17 were treated as ordered and an equally weighted parsimony analysis was performed using TNT (Tree analysis using New Technology) version 1.5^48^. An implicit enumeration tree search was conducted to provide an exact solution given the number of taxa in the matrix allowed this option. The strict consensus tree was generated from the most parsimonious trees obtained. Nodal support was evaluated using Bremer support and parsimony jackknifing in TNT. We additionally conducted a parsimony implied weight analysis in TNT^48^ and a Bayesian analysis using Mr. Bayes using the Mkv model^49^ to test if the results obtained were sensitive to the choice of phylogenetic analytical procedures. See Supplementary Information for further details. Osteological terminology follows^50^.

## Supplementary Information

Supplementary Information.

## References

[1] del Hoyo, J., Elliott, A. & Sargatal, J. *Handbook of the Birds of the World. Vol. 6. Mousebirds to Hornbills* (Lynx Edicions, 2001).

[2] Wetmore A, Miller WW (1926). The revised classification for the fourth edition of the AOU Check-list. Auk.

[3] Prum RO, Berv JS, Dornburg A, Field DJ, Townsend JP, Lemmon EM, Lemmon AR (2015). A comprehensive phylogeny of birds (Aves) using targeted next-generation DNA sequencing. Nature.

[4] Prum RO, Berv JS, Dornburg A, Field DJ, Townsend JP, Lemmon EM, Lemmon AR (2016). A comprehensive phylogeny of birds (Aves) using targeted next-generation DNA sequencing. Nature.

[5] Brodkorb P (1970). An eocene puffbird from wyoming. Contrib. Geol..

[6] Houde P, Olson SL (1992). A radiation of coly-like birds from the Eocene of North America. Contrib. Sci. Nat. Hist. Mus. Los Angeles Co..

[7] Ksepka DT, Clarke JA (2010). *Primobucco mcgrewi* (Aves: Coracii) from the Eocene Green River Formation: New anatomical data from the earliest constrained record of stem rollers. J. Vert. Paleontol..

[8] Clarke JA, Ksepka DT, Smith NA, Norell MA (2009). Combined phylogenetic analysis of a new North American fossil species confirms widespread Eocene distribution for stem rollers (Aves, Coracii). Zool. J. Linn. Soc..

[9] Bourdon E, Kristoffersen AV, Bonde N (2016). A roller-like bird (Coracii) from the Early Eocene of Denmark. Sci. Rep..

[10] Mayr G, Mourer-Chauviré C (2000). Rollers (Aves: Coraciiformes s.s.) from the Middle Eocene of Messel (Germany) and the Upper Eocene of the Quercy (France). J. Vert. Paleontol..

[11] Tambussi, C.P. & Degrange, F.J. *South American and Antarctic Continental Cenozoic Birds*. (SpringerBriefs in Earth System Sciences, 2013).

[12] Aragón E, Mazzoni MM (1997). Geología y estratigrafía del complejo volcánico piroclástico del río Chubut medio (Eoceno), Chubut, Argentina. RAGA.

[13] Wilf P (2003). High plant diversity in Eocene South America: Evidence from Patagonia. Science.

[14] Wilf P (2005). Eocene plant diversity at Laguna del Hunco and Río Pichileufú, Patagonia, Argentina. Am. Nat..

[15] Wilf P, Nixon KC, Gandolfo MA, Cúneo NR (2019). Eocene Fagaceae from Patagonia and Gondwanan legacy in Asian rainforests. Science.

[16] Dolgopol de Sáez M (1941). Noticias sobre peces fósiles argentinos, Siluroideos Terciarios del Chubut. Not. Mus. La Plata.

[17] Casamiquela RM (1960). Un pipoideo fósil de Patagonia. Rev. Mus. La Plata Pal..

[18] Báez AM, Trueb L (1997). Redescription of the Paleogene *Shelania pascuali* from Patagonia and its bearing on the relationships of fossil and recent pipoid frogs. Univ. Kans. Nat. Hist. Mus. Sci. Pap..

[19] Gosses J, Carroll AR, Bruck BT, Singer BS, Jicha BR, Aragón E, Walters AP, Wilf P (2020). Facies interpretation and geochronology of diverse Eocene floras and faunas, northwest Chubut Province, Patagonia, Argentina. Geol. Soc. Am. Bull..

[20] Mourer-Chauviré C (1985). Les Todidae (Aves, Coraciiformes) des Phosphorites du Quercy (France). P. K. Ned. Akad. Wetensc. B.

[21] Mayr G, Knopf CW (2007). A tody (Alcediniformes: Todidae) from the early Oligocene of Germany. Auk.

[22] Mayr G (2008). The Madagascan “Cuckoo-roller” (Aves: Leptosomidae) is not a roller—notes on the phylogenetic affinities and evolutionary history of a “living fossil”. Acta Ornithol..

[23] Mayr G, Mourer-Chuaviré C (2000). Rollers (Aves: Coraciiformes s.s.) from the Middle Eocene of Messel (Germany) and the Upper Eocene of the Quercy (France). J. Vert. Paleontol..

[24] Mayr G, Mourer-Chauviré C, Weidig I (2004). Osteology and systematic position of the Eocene Primobucconidae (Aves, Coraciiformes sensu stricto), with first records from Europe. J. Syst. Palaeontol..

[25] Mayr G (2009). Paleogene Fossil Birds.

[26] Mayr G, Mourer-Chauviré C (2005). A specimen of *Parvicuculus* Harrison & Walker 1977 (Aves: Parvicuculidae) from the early Eocene of France. B. Brit. Ornithol. Club.

[27] Cracraft J (1971). The relationships and evolution of the Rollers: Families Coraciidae, Brachypteraciidae, and Leptosomatidae. Auk.

[28] Mourer-Chauviré, C., Peyrouse, J. & Hugueney, M. A new roller (Aves: Coraciiformes s. s.: Coraciidae) from the Early Miocene of the Saint-Gérand-le-Puy area, Allier, France in Proceedings of the 8th International Meeting of the Society of Avian Paleontology and Evolution (eds. Göhlich, U.B. & Kroh, A.) 81–92 (Verlag Naturhistorisches Museum Wien, 2013).

[29] Ksepka DT, Stidham TA, Williamson TE (2017). Early Paleocene landbird supports rapid phylogenetic and morphological diversification of crown birds after the K-Pg mass extinction. PNAS.

[30] Field DJ, Hsiang AY (2018). A North American stem turaco, and the complex biogeographic history of modern birds. BMC Evol. Biol..

[31] Ksepka DT, Clarke JA (2010). New fossil mousebird (Aves: Coliiformes) with feather preservation provides insight into the ecological diversity of an Eocene North American avifauna. Zool. J. Linn. Soc..

[32] Ksepka DT, Clarke JA (2009). Affinities of *Palaeospiza bella* and the phylogeny and biogeography of mousebirds (Coliiformes). Auk.

[33] Olson, S.L. A new family of primitive landbirds from the Lower Eocene Green River Formation of Wyoming. In *Papers in Avian Paleontology honoring Pierce Brodkorb* (ed. Campbell Jr, K.E.) 137–160 (Natural History Museum of Los Angeles County, 1992).

[34] Claramunt S, Cracraft J (2015). A new time tree reveals Earth history’s imprint on the evolution of modern birds. Sci. Adv..

[35] McCullough JM, Moyle RG, Smith BT, Andersen MJ (2019). A Laurasian origin for a pantropical bird radiation is supported by genomic and fossil data (Aves: Coraciiformes). Proc. R. Soc. B.

[36] Mayr G (1998). “Coraciiforme” und “Piciforme” Kleinvögel aus dem Mittel-Eozän der Grube Messel (Hessen, Deutschland). Cour. Forsch. Inst. Senckenberg.

[37] Andersen MJ, McCullough JM, Mauck WM, Smith BT, Moyle RG (2018). A phylogeny of kingfishers reveals an Indomalayan origin and elevated rates of diversification on oceanic islands. J. Biogeogr..

[38] Smith ME, Carroll AR, Singer BS (2008). Synoptic reconstruction of a major ancient lake system: Eocene Green River Formation, western United States. GSA Bull..

[39] Ericson PGP (2012). Evolution of terrestrial birds in three continents: Biogeography and parallel radiations. J. Biogeogr..

[40] Oliveira, F.B., Cassola Molina, E. & Marroig, G. Paleogeography of the South Atlantic: A route for primates and rodents into the New-World? In *South American Primates, Developments in Primatology: Progress and Prospects* (eds. Garber, P.A., Estrada, A., Bicca-Marques, J.C., Heymann, E.W. & Strier, K.B.) 55–68 (Springer Science, New York, 2010).

[41] Ezcurra MD, Agnolín FL (2012). A new global palaeobiogeographical model for the Late Mesozoic and Early Tertiary. Syst. Biol..

[42] Antoine PO (2012). Middle Eocene rodents from Peruvian Amazonia reveal the pattern and timing of caviomorph origins and biogeography. Proc. R. Soc. Lond. B.

[43] Bond M (2015). Eocene primates of South America and the African origins of New World monkeys. Nature.

[44] Mourer-Chauviré C, Tabuce R, Mahboubi M, Adaci M, Bensalah M (2011). A Phororhacoid bird from the Eocene of Africa. Naturwissenschaften.

[45] Mayr G, Alvarenga H, Mourer-Chauviré C (2011). Out of Africa: Fossils shed light on the origin of the hoatzin, an iconic Neotropic bird. Naturwissenschaften.

[46] Carranza S, Arnold EN, Mateo JA, López-Jurado LF (2000). Long- distance colonization and radiation in gekkonid lizards, Tarentola (Reptilia: Gekkonidae), revealed by mitochondrial DNA sequences. Proc. R. Soc. Lond. B..

[47] Vidal N, Azvolinsky A, Cruaud C, Hedges SB (2008). Origin of tropical American burrowing reptiles by transatlantic rafting. Biol. Lett..

[48] Goloboff PA, Catalano SA (2016). TNT version 1.5, including a full implementation of phylogenetic morphometrics. Cladistics.

[49] Ronquist F, Teslenko M, Van Der Mark P, Ayres DL, Darling A, Hohna S, Larget B, Liu L, Suchard MA, Huelsenbeck JP (2012). MrBayes 3.2: Efficient bayesian phylogenetic inference and model choice across a large model space. Syst. Biol..

[50] Baumel, J.J. & Witmer, L.M. 1993. Osteologia. In *Handbook of Avian Anatomy: Nomina Anatomica Avium* (eds. Baumel, J.J., King, A.S., Breazile, J.E., Evans, H.E. & Vanden Berge, J.C.) 45–132 (Cambridge, 1993).

