# Supplementary material for: Unexpected larger distribution of paleogene stem-rollers (AVES, CORACII): new evidence from the Eocene of Patagonia, Argentina
Source: Sci Rep. 2021 Jan 14;11:1363. doi: 10.1038/s41598-020-80479-8 (PMC7809110; doi:10.1038/s41598-020-80479-8)
Supplement: Supplementary file 1 — Supplementary Information. [file 41598_2020_80479_MOESM1_ESM.docx]

**Supplementary Information to: UNEXPECTED LARGER DISTRIBUTION OF PALEOGENE STEM-ROLLERS (AVES, CORACII): NEW EVIDENCE FROM THE EOCENE OF PATAGONIA, ARGENTINA**

Federico J. Degrange^1*^, Diego Pol^2^, Pablo Puerta^2^ & Peter Wilf^3^

^1^Centro de Investigaciones en Ciencias de la Tierra (CICTERRA), UNC, CONICET, Avenida Vélez Sársfield 1611, X5016GCA, Córdoba, Argentina. *fjdino@gmail.com*

^2^Museo Paleontológico Egidio Feruglio-CONICET. Av. Fontana 140, U9100GYO, Trelew, Chubut, Argentina. *dpol@mef.org.ar*, *[ppuerta@mef.org.ar](mailto:ppuerta@mef.org.ar)*

^3^Department of Geosciences and Earth and Environmental Systems Institute, Pennsylvania State University, University Park, Pennsylvania 16802, United States. *pwilf@psu.edu*

**1. Phylogenetic analysis**

The phylogenetic analysis used in this contribution was based on an equally weighted parsimony analysis using TNT [1]. The two most parsimonious trees recovered in the implicit enumeration tree search had 205 steps in length and a consistency index CI=0.424 and a retention index RI=0.655. Below we include the results of the Bremer support and jackknife analysis (Figs. S1, S2). In addition to these two support analyses we tested positioning *Ueekenkcoracias* in other clades of Picocoraciae using the constrained tree searches through the *force* command to find out how many extra steps were required to place the new species in different placements of the phylogenetic tree.

In addition to this approach, we have also run the same dataset using other methods that use alternative optimality criteria. For instance, analyses of the same dataset parsimony under implied weights [2] yielded trees in which *Ueekenkcoracias* is placed in the same phylogenetic position, as the basalmost stem-roller (Fig. S3). This result is obtained under a broad range of weighting functions (constant k ranging between 3 and 15).

Similarly, we have run a Bayesian analysis using MrBayes 3.2 [3] under the Mkv model for 10,000,000 generations. Four independent runs were performed, setting the coding options as variable or informative and the rates as equal or gamma distributed. All analyses conducted coincide in in the phylogenetic position *Ueekenkcoracias* as the basalmost stem-roller (Fig. S4). The posterior probability values for Coracii (including *Ueekenkcoracias*) were always above 80% (Fig. S4). However other nodes within the clade are lower, such as the group clustering all other Coracii with the exception of *Ueekenkcoracias* (e.g., 56%; Fig. S4). This relatively low posterior probability value is in concordance with the low support values of this nodes obtained in the parsimony analysis (see below).

**2. Data matrix**

*Tyto* 000000110000100000000000000001000010?00001000000000000001000000000010010000000

*Colius* 100000100001000000001000010100000010?0100000000010000001000?010000100000000010

*Harpactes* 000000100000002100000100000000000000?01000000001000000000000100000001000001001

*Septencoracias* ?00?000????0????0??00???00????000001000001000?0??111?0100???00100?011011100111

*Primobucco* 10??0001?0??101?0??1?000???000000001000001000????11?101?0???1???0?0?1011100011

*Eocoracias* ?0???0?100??201?0???00?0??00?0001001?00?00001????11??01?0???0???00??10???00000

*Paracoracias* 11??001100??201?0??10?????0?0000000??00?00001????1???01?0???10??0????01?1?0101

*Geranopterus* ??0?????????21??????????1000??0??01101?0??00?????1?1101???????100101101110??01

*Coracias* 111101111010211010010100110000000011110000001000011110100100101101011011100101

*Atelornis* 11110011100021100001010010000000111111000000000001111010010?101101101010100100

*Baryphthengus* 00010010?100101111000001100100001010?10000000000011000110111100010000000010100

*Todus* 1001001010000011110000011?0100101010?1001000000001100011011110001010?000?10100

*Merops* 001000000000001110000001110010001011010000000000011000110101000000101001011100

*Alcedo* 001110111010000110000001101000101010?10000001000011100110101101010101001011100

*Dacelo* 001110111010101110000001100000001010?10000000000011100110101100010101011011100

*Upupa* 1001111??1?1001020101010011011111120?11110011101101000101100010000100100010000

*Phoeniculus* 1001111??1?1000020101011011011111120?11110011101111000111100010000100100010100

*Bucorvus* 1001011000?1101020001010010001101020?10000000101011000100100010010000100010000

*Nystalus* 111100110011201010101210001000001110?1000010000001100100000001001010?100000100

*Pteroglossus* 0200011??1?1101020001021010100112010?10000100010010002000000010110100100010100

*Picoides* 0000000??1?110101?001021110100112110?10000100010100002001000111110100100010100

*Ueekenkcoracias* ????????????????????????????????????????????????0??1??000?????????????011?00??

**3. Supplementary Figures**

**Figure S1. Bremer Support values.** This analysis was conducted using the script Bremer.run in TNT [1] for the equally weighted parsimony analysis.


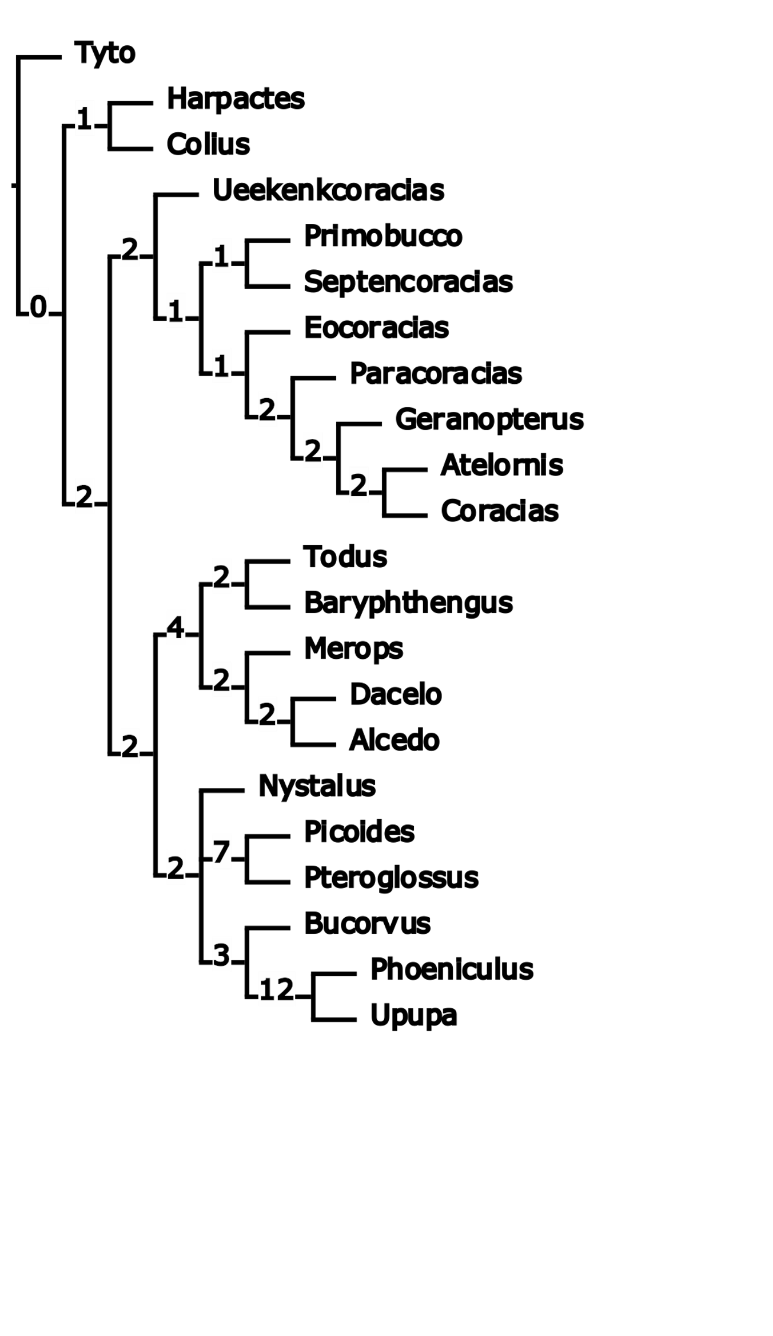


**Figure S2. Jackknife Support values.** This analysis was conducted using the command *resample* in TNT [1] for the equally weighted parsimony analysis. Values shown by the nodes represent the GC and absolute frequencies.


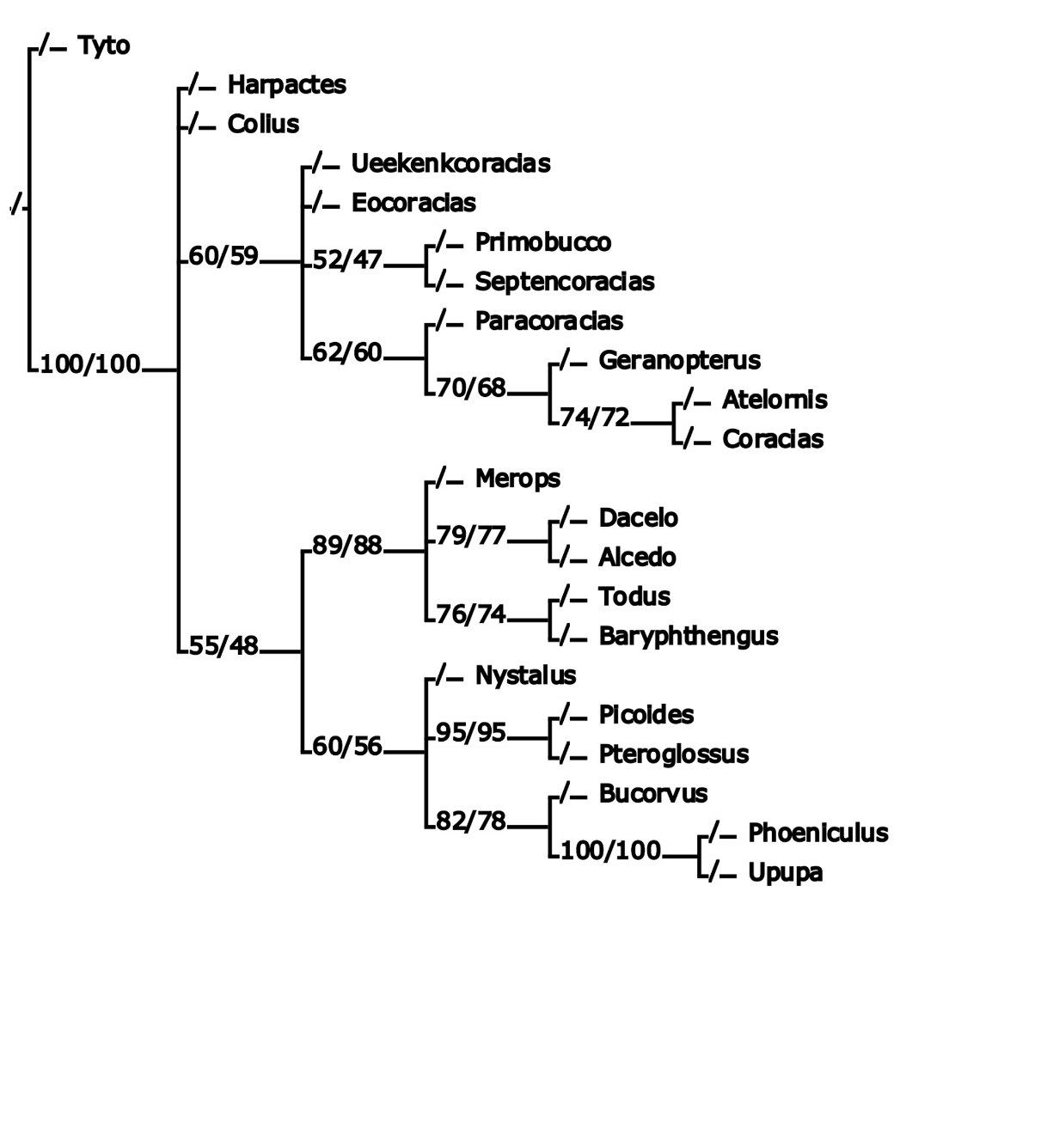


**Figure S3. Implied weights analysis.** This tree was obtained using a K value of 5 in TNT [1], similar results were obtained for other weighting constant values.


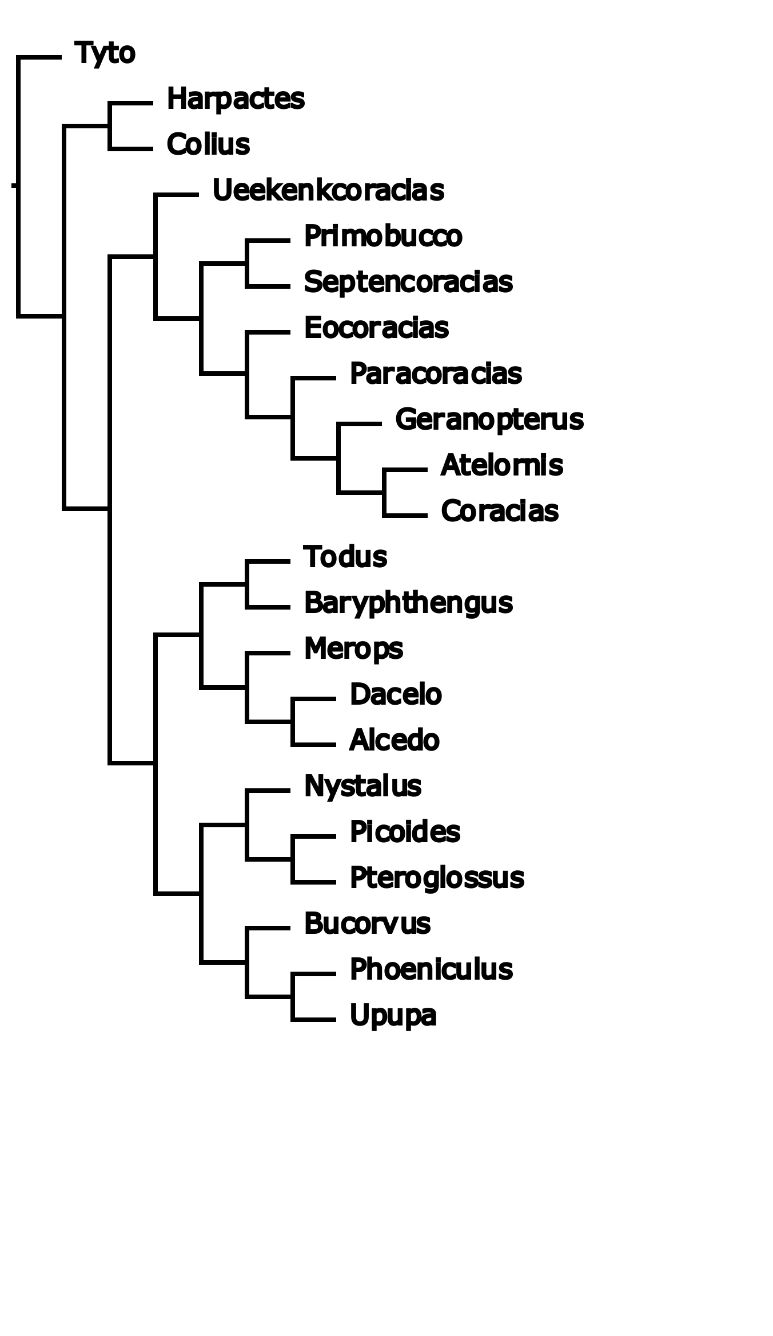


**Figure S4. Bayesian analysis.** Maximum credibility tree of the analysis conducted in Mr. Bayes setting the coding options as informative and the rates as gamma distributed.


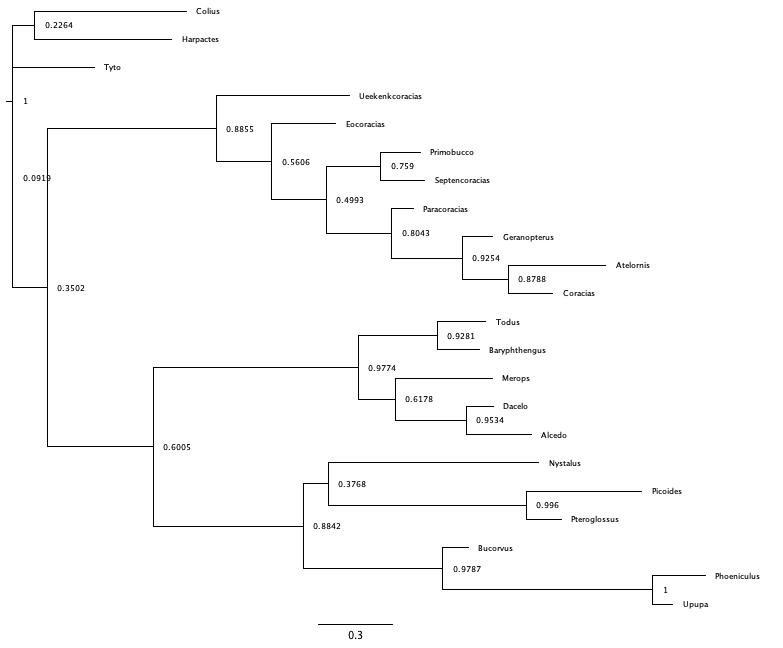


**4. Supplementary references**

1. Goloboff, P.A. & Catalano, S.A. TNT version 1.5, including a full implementation of phylogenetic morphometrics. *Cladistics* **32**, 221–238 (2016).

2. Goloboff P.A. Estimating character weights during tree search. *Cladistics* **9**, 83–91 (1993).

3. Ronquist, F., Teslenko, M., Van Der Mark, P. Ayres, D.L., Darling, A., Hohna, S., Larget, B., Liu, L., Suchard, M.A. & Huelsenbeck, J.P. MrBayes 3.2: Efficient bayesian phylogenetic inference and model choice across a large model space. *Syst. Biol.* **61**, 539–542 (2012).
